# Adenosine and γ-aminobutyric acid partially regulate metabolic and ventilatory responses of Damaraland mole-rats to acute hypoxia

**DOI:** 10.1242/jeb.246186

**Published:** 2023-10-06

**Authors:** Maiah E. M. Devereaux, Matthew E. Pamenter

**Affiliations:** ^1^ Department of Biology, University of Ottawa, Ottawa, ON K1N 6N5, Canada; ^2^ University of Ottawa Brain and Mind Research Institute, Ottawa, ON K1H 8M5, Canada

**Keywords:** Adenosine receptors, GABA receptors, Bicuculline, Aminophylline, Hypoxic ventilatory response, Thermoregulation, Metabolic suppression

## Abstract

Fossorial Damaraland mole-rats (*Fukomys damarensis*) mount a robust hypoxic metabolic response (HMR) but a blunted hypoxic ventilatory response (HVR) to acute hypoxia. Although these reflex physiological responses have been described previously, the underlying signalling pathways are entirely unknown. Of particular interest are contributions from γ-aminobutyric acid (GABA), which is the primary inhibitory neurotransmitter in the nervous system of most adult mammals, and adenosine, the accumulation of which increases during hypoxia as a breakdown product of ATP. Therefore, we hypothesized that GABAergic and/or adenosinergic signalling contributes to the blunted HVR and robust HMR in Damaraland mole-rats. To test this hypothesis, we injected adult animals with saline alone (controls), or 100 mg kg^−1^ aminophylline or 1 mg kg^−1^ bicuculline, to block adenosine or GABA_A_ receptors, respectively. We then used respirometry, plethysmography and thermal RFID probes to non-invasively measure metabolic, ventilator and thermoregulatory responses, respectively, to acute hypoxia (1 h in 5 or 7% O_2_) in awake and freely behaving animals. We found that bicuculline had relatively minor effects on metabolism and thermoregulation but sensitized ventilation such that the HVR became manifest at 7% instead of 5% O_2_ and was greater in magnitude. Aminophylline increased metabolic rate, ventilation and body temperature in normoxia, and augmented the HMR and HVR. Taken together, these findings indicate that adenosinergic and GABAergic signalling play important roles in mediating the robust HMR and blunted HVR in Damaraland mole-rats.

## Teaser

**Summary:** Damaraland mole-rats use inhibitory signalling mechanisms to regulate robust physiological responses to acute hypoxia.

## INTRODUCTION

The initial physiological reflex response of most adult mammals to inhalation of hypoxic gas is to increase ventilation (Pamenter and Powell, 2016; Powell et al., 1998). This is referred to as the hypoxic ventilatory response (HVR) and is one of the primary mechanisms through which O_2_ supply to the body can be increased in hypoxic environments. In most adult mammals, the HVR is biphasic, consisting of a sharp increase in ventilation following the initial detection of inhaled hypoxia, followed by a decrease after several minutes of sustained hypoxic exposure to a new baseline level that remains above normoxic ventilation. Neonatal mammals also exhibit a biphasic HVR; however, neonatal ventilation is often reduced below normoxic levels during the second phase of the HVR (Mortola et al., 1989), which likely contributes to energy savings in hypoxia.

The signalling mechanisms that regulate the adult mammalian HVR are well understood in non-fossorial mammals. In most such species, excitatory glutamatergic signalling mediates the first phase of the HVR and also underlies the increase in ventilatory tone during the second phase*,* whereas inhibitory γ-aminobutyric acid (GABAergic) signalling plays a central role in the decline of ventilation in the second phase of the HVR, relative to the initial peak of the first phase (Pamenter and Powell, 2016; Teppema and Dahan, 2010). In neonates, the glutamatergic system is not fully developed, and instead, the GABAergic system dominates neurotransmission (Wong-Riley et al., 2019), resulting in a net inhibitory shift of the neonatal HVR. A similar system appears to regulate the HVR of eusocial naked mole-rats, which are among the most hypoxia-tolerant mammals and putatively experience intermittent hypoxia in their crowded underground burrows (Buffenstein et al., 2022; Chung et al., 2016; Pamenter, 2022; Pamenter et al., 2019, 2018). Specifically, and unlike in all other adult mammals studied (but similar to neonates from non-fossorial mammalian species), modulation of glutamate receptors has no impact on the HVR of naked mole-rats exposed to 7% O_2_ (Dzal et al., 2019). Instead, antagonism of GABA receptors abrogates the HVR in this species (Chung et al., 2016), suggesting a central role for GABA receptors in the control of the HVR in a fossorial mammal.

Conversely, few studies have explored the control of metabolic responses to hypoxia in adult mammals, presumably because a robust hypoxic metabolic response (HMR) is not a commonly observed strategy in hypoxia-intolerant adult mammals, which have received the lion's share of research attention in this field. As a result, the mechanisms that regulate the HMR are poorly understood, and knowledge in this area is particularly lacking in fossorial species. However, this question has also recently received some attention in naked mole-rats: in response to hypoxia, naked mole-rats drastically reduce their metabolic rate (Pamenter et al., 2015, 2019), in part owing to a rapid decrease in thermogenesis (Cheng et al., 2021; Kirby et al., 2018; Vandewint et al., 2019). Unlike in other adult mammals, the naked mole-rat HMR is mediated by inhibitory signaling, with adenosine playing a key role (Chung et al., 2016; Dzal et al., 2019; Pamenter, 2022; Pamenter et al., 2015). Adenosine is a breakdown product of ATP hydrolysis and the concentration of adenosine in the blood increases during periods of hypoxia in most species (Buck, 2004; Phillis et al., 1992; Saito et al., 1999); therefore, it is a promising candidate to regulate physiological responses to hypoxia.

Damaraland mole-rats (*Fukomys damarensis*) are close phylogenetic cousins of naked mole-rats, are the only other eusocial mammal, and also live in large, densely populated underground colonies (Thomas et al., 2016). The physiological response of Damaraland mole-rats to acute hypoxia has recently been described and is like that of naked mole-rats: they have a blunted HVR and a robust HMR (Ivy et al., 2020). Therefore, Damaraland mole-rats may also have similar control pathways underlying these responses. Unfortunately, nothing is known about the signalling mechanisms that control physiological responses to hypoxia in this species.

Given the role for GABAergic and adenosinergic signalling in mediating metabolic and ventilatory adaptations to hypoxia in naked mole-rats, and the generally inhibitory tone of physiological responses to acute hypoxia in Damaraland mole-rats, we hypothesized that the same signalling pathways regulate the hypoxic metabolic and ventilatory responses in Damaraland mole-rats. To test this hypothesis, we measured metabolism, ventilation and thermoregulation in freely behaving Damaraland mole-rats exposed to normoxia (21% O_2_) and then acute hypoxia (1 h in 7 or 5% O_2_), with and without intraperitoneal injections of the adenosine receptor antagonist aminophylline or the GABA_A_ receptor antagonist bicuculline.

**List of symbols and abbreviations**

ACR: air convection requirement
*f*_R_: breathing frequency
GABA: gamma-amino butyric acid
HMR: hypoxic metabolic response
HVR: hypoxic ventilatory response
RER: respiratory exchange ratio
*T*_b_: body temperature
*V̇*_E_: minute ventilation
*V̇*_CO_2__: carbon dioxide production
*V̇*_O_2__: oxygen consumption rate
*V*_T_: tidal volume

## MATERIALS AND METHODS

### Animals

Damaraland mole-rats [*Fukomys damarensis* (Ogilby 1838)] were bred at the University of Ottawa group-housed in interconnected multi-cage systems at 22°C in 21% O_2_ and 0.04% carbon dioxide (CO_2_) and 50% humidity with a 12 h:12 h light:dark cycle. Animals were fed fresh tubers, vegetables, fruit and Pronutro cereal supplement *ad libitum*. The age of our experimental animals was selected based on a growth study of Damaraland mole-rats, which reported that this species reaches 90% of their full size by 600 days post-birth (Thorley and Clutton-Brock, 2019). Neural dimorphisms exist dependent on breeding status such that breeding animals have greater development in certain areas of the brain relative to subordinates of both sexes (Anyan et al., 2011), which may have unpredictable impacts on the neural control of physiological responses to hypoxia. As such, the queen and breeding male from our colony were omitted from this study.

### Experimental ethics and design

All experimental procedures were approved by the University of Ottawa's Animal Care Committee (protocol no. 2535) and conducted in accordance with the Animals for Research Act and other regulations of the Canadian Council on Animal Care. Experiments were performed during the daylight portion of the animals' daily light:dark cycle, and the experimental time within this window was randomized for each individual animal and experimental protocol to remove any bias induced by circadian rhythms. Briefly, 10 adult subordinate Damaraland mole-rats (5 males, 5 females, age 1.5–3 years; 208.1±12.3 g, mean±s.e.m.) were subjected to each of five experimental conditions, consisting of exposure to 1 h of normoxia followed by intraperitoneal injections of: saline alone (controls), or aminophylline (to block adenosine receptors; 100 mg kg^−1^) (Barros et al., 2006; Martin, 1946; Pamenter et al., 2015) or bicuculine (to block GABA_A_ receptors; 1 mg kg^−1^) (Beart and Johnston, 1972; Chung et al., 2016; Curtis et al., 1970a,b). Animals were then replaced in normoxic conditions for 1 h to assess the impact of the drug on the physiological parameters of interest, and were finally then exposed to either 7 or 5% O_2_ for 1 h in random order (protocols 1 and 2, respectively). The doses and half-lives of both drugs are sufficient to ensure efficacy *in vivo* for at least 3 h post-injection (Gale and Casu, 1981; Lohmann and Miech, 1976; Ueno et al., 1997). Aminophylline had major effects on metabolic rate (see Results), and animals treated with this drug were unable to tolerate exposure to 5% O_2_. Therefore, the impact of aminophylline in hypoxia was only tested in 7% O_2_ and the sample size for this dataset was reduced relative to the other experimental groups owing to animal deaths in pilot trials in 5% O_2_. Saline experiments in both 7 and 5% O_2_ are also presented in Devereaux et al. (2023). Animals were not fasted prior to experimental trials and all animals were exposed to all six experimental paradigms (3× injected treatments in two different hypoxic conditions) in random order. Animals were permitted a minimum of 1 week of rest between experiments to reduce any confounding effects of previous hypoxic exposures.

For each experiment, animals were individually placed unrestrained in a 1.0 l Plexiglass experimental chamber held at 22°C in normoxia (21% O_2_/0.04% CO_2_) until O_2_ consumption reached steady state for a minimum of 20 min. For the purposes of these experiments, we defined steady state as a period where the concentration of O_2_ in the excurrent air did not fluctuate by more than 0.2% for a minimum of 15 min. Following this normoxic control period, animals were removed from the experimental chamber and injected intraperitoneally with either saline or one of the two drugs. Drugs were diluted to inject animals with a standard bolus of 2.5 µl g^−1^ of body mass and drug dosages were based on previous experiments performed on naked mole-rats in our lab, which were in turn based on studies in other small rodents (see references above). Dosages were updated for the Damaraland mole-rats on an as-needed basis. Animals must have been awake and not exhibiting signs of physical stress (e.g. haunching, scratching or licking injection site, irregular or strained breathing patterns, etc.) post-injection before they were subjected to experimentation.

After injection, animals were placed back into normoxia until O_2_ consumption reached steady state. This was done to control for any effects the drugs may have had on metabolic or ventilatory parameters while the animals were still breathing normoxic air, and to account for stress following the injection and related handling. Finally, O_2_ was lowered to 7% or 5%, taking 15–30 min to achieve the new hypoxic equilibrium in the experimental chamber and until metabolic parameters once again reached steady state. Our lab has previously conducted experiments in naked mole-rats using these same drug treatments in a 7% O_2_ hypoxic exposure. Therefore, we chose this O_2_ level for our experiments to permit simple comparison between the two species. However, in preliminary studies we did not observe a ventilatory response in 7% O_2_ (protocol 1). Therefore, we repeated all experiments at a deeper level of hypoxia (5% O_2_; protocol 2) to better assess the effect of each drug on ventilation. Although Damaraland mole-rats have been studied in as low as 3% O_2_, pilot trials determined this was only tolerable for a maximum of 35 min with saline, and for less time in certain drug trials. Thus, 5% O_2_ was determined to be the lowest safe O_2_ level for the animals and we did not test deeper levels of hypoxia. Each experimental stage lasted approximately 1 h.

To maximize the sensitivity and resolution of metabolic rate measurements, gases were continuously supplied at 0.4 l min^−1^. As a result, animal-induced changes in the concentrations of O_2_ and CO_2_ would have altered the gas composition in the experimental chamber, reducing O_2_ by ∼0.4–2.0% and increasing CO_2_ by ∼0.1–1.0%, depending on their metabolic activity. However, standard practices in this field are based largely on studies in hypoxia-intolerant animals, such as mice, dogs and rats, which are considerably more sensitive to smaller changes in atmospheric O_2_ and CO_2_. Conversely, Damaraland mole-rats live at mild altitude in nature, in which atmospheric air is ∼18.5% O_2_, and their metabolic and ventilatory responses to hypoxia and hypercapnia are not activated until well beyond 19% O_2_ or 1% CO_2_ (Ivy et al., 2020; Zhang and Pamenter, 2019a,b). Therefore, it is unlikely that this affected our evaluation of the regulation of the HVR and HMR in this species.

### Respirometry

During experimentation, the animal chamber was sealed and continuously ventilated with gas mixtures, set to the desired fractional gas composition by calibrated rotameters (Praxair, Mississauga, ON, Canada). Incurrent air was bubbled in distilled H_2_O prior to entering the animal chamber. Excurrent air passed through an RH-300 Water Vapour Pressure Analyzer to measure humidity, and the average incurrent gas humidity was 94.5±3.1%, which is consistent with previous experiments using similar equipment in our laboratory (Clayson et al., 2020). Excurrent gas passed through a desiccant medium before entering the O_2_ and CO_2_ analyzers. The final 10-min period of stable activity from each experimental stage was used for data analysis. To determine metabolic rate, eqns 10.6 and 10.7 from Lighton (2008) were used to calculate the rate of O_2_ consumption (*V̇*_O_2__, ml min^−1^ kg^−1^) and the rate of CO_2_ production (*V̇*_CO_2__, ml min^−1^ kg^−1^), respectively:
(1)

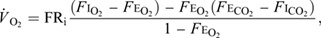

(2)

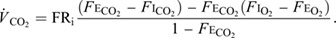


In these equations, FR_i_ is the incurrent flow rate (ml min^−1^), *F*i_O_2__ and *F*i_CO_2__ are the fractional concentrations of incurrent O_2_ and CO_2_ of dry gas, respectively, and *F*e_O_2__ and *F*e_CO_2__ are the fractional concentrations of excurrent O_2_ and CO_2_ from the experimental chamber, respectively (Lighton, 2008). Respiratory exchange ratios (RERs) were calculated as *V̇*_CO_2__/*V̇*_O_2__.

### Thermal measurements

Body temperature (*T*_b_, °C) was recorded non-invasively every 10 min throughout all experiments using an RFID microchip reader (Allflex USA Inc., Dallas, TX, USA) to scan previously implanted subcutaneous RFID microchips (Destron Fearing) along the back flank of the animal. Chamber temperature was recorded during each inflow period using a custom thermocouple.

### Whole-body plethysmography

Attached to the experimental chamber was an identical second chamber that acted as a reference chamber. Continuous monitoring by a differential pressure transducer connected between the two chambers amplified small pressure fluctuations in the experimental chamber, allowing us to detect and measure breaths at 1000 Hz. Before each trial, the transducer was calibrated by injecting six known volumes of air (0.1, 0.2, 0.3, 0.4, 0.5 and 0.6 ml) 10 times into the experimental chamber. Injections were performed with continuous airflow through the pressure-sealed system at the same respiratory frequency (*f*_R_, breaths min^−1^) the animal was observed to have under normoxic/normocapnic conditions. To calculate tidal volume (*V*_T_, ml kg^−1^) and *f*_R_, five breath sets consisting of a minimum of 10 consecutive and clearly defined breaths within the same 10 min period as that used for metabolic rate calculations were analyzed. The Drorbaugh and Fenn (1955) equation was used to calculate *V*_T_:
(3)

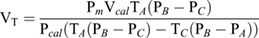


The average oscillation height was taken from each breath set, representing the average total pressure deflection of a breath (*P*_m_). *P*_cal_ (V) and *V*_cal_ (µl) are the pressure deflection and volume of a known calibrated volume, respectively. The average oscillation height of each calibration set was plotted against its respective volume and used to create a linear relationship. The point on this line representing 0.2 ml was chosen as *P*_cal_ and *V*_cal_. *T*_A_ is the body temperature of the animal (K) and *T*_C_ is the temperature of the chamber (K), both recorded at the end of the 10 min period. *P*_B_ is the barometric pressure in the lab (mmHg) as measured by the O_2_ analyzer. *P*_A_ is the vapour pressure of water at the animal's body temperature (mmHg) and P_C_ is the partial pressure of water vapour (mmHg) in the incurrent gas stream (Drorbaugh and Fenn, 1955). *P*_A_ was calculated using relative humidity (%) of excurrent air, animal temperature (°C) and barometric pressure (kPa). *P*_C_ used relative humidity (%) of incurrent air, chamber temperature (°C) and barometric pressure (kPa). The same breath samples were used to calculate *f*_R_. Minute ventilation (*V̇*_E_) was calculated as the product of *f*_R_ and *V*_T_. The air convection requirements (ACRs) for O_2_ and CO_2_ were calculated as the quotient of *V̇*_E_ and *V̇*_O_2__ or *V̇*_CO_2__, respectively.

### Analysis

Ventilatory and metabolic data were collected using LabChart software (ADInstruments, Colorado Springs, CO, USA) and analysed in PowerLab (ADInstruments). Statistical analysis was performed using commercial software (Prism v.9.2.0, GraphPad Software Inc., La Jolla, CA, USA). Responses to hypoxia and changes mediated by aminophylline or bicuculline administration were not different between sexes and so these data were pooled in our analysis. *P*<0.05 was considered the threshold for statistical significance. Data are presented in box and whisker plots. Aminophylline and bicuculline target pathways that do not directly interact, and which were not inherently linked by our hypothesis; therefore, we compared the results of drug trials directly with saline controls only and not with each other. Statistical significance was evaluated using a two-way repeated-measures analysis of variance (RM-ANOVA) to test for interactions between normoxia and hypoxia (7 or 5% O_2_), or drug treatments. Tukey's or Šidák's multiple comparisons tests were performed on each dependent variable to determine significance. Statistical significance between hypoxia-mediated changes in each variable were analyzed using Student’s *t*-tests.

## RESULTS

Note that for all datasets, statistical analysis of control (saline) experiments is presented in the companion paper to this study (Devereaux et al., 2023) and is not duplicated here.

### Metabolism and thermoregulation are minimally impacted by GABA_A_ receptor antagonism

**Fig. 1.**
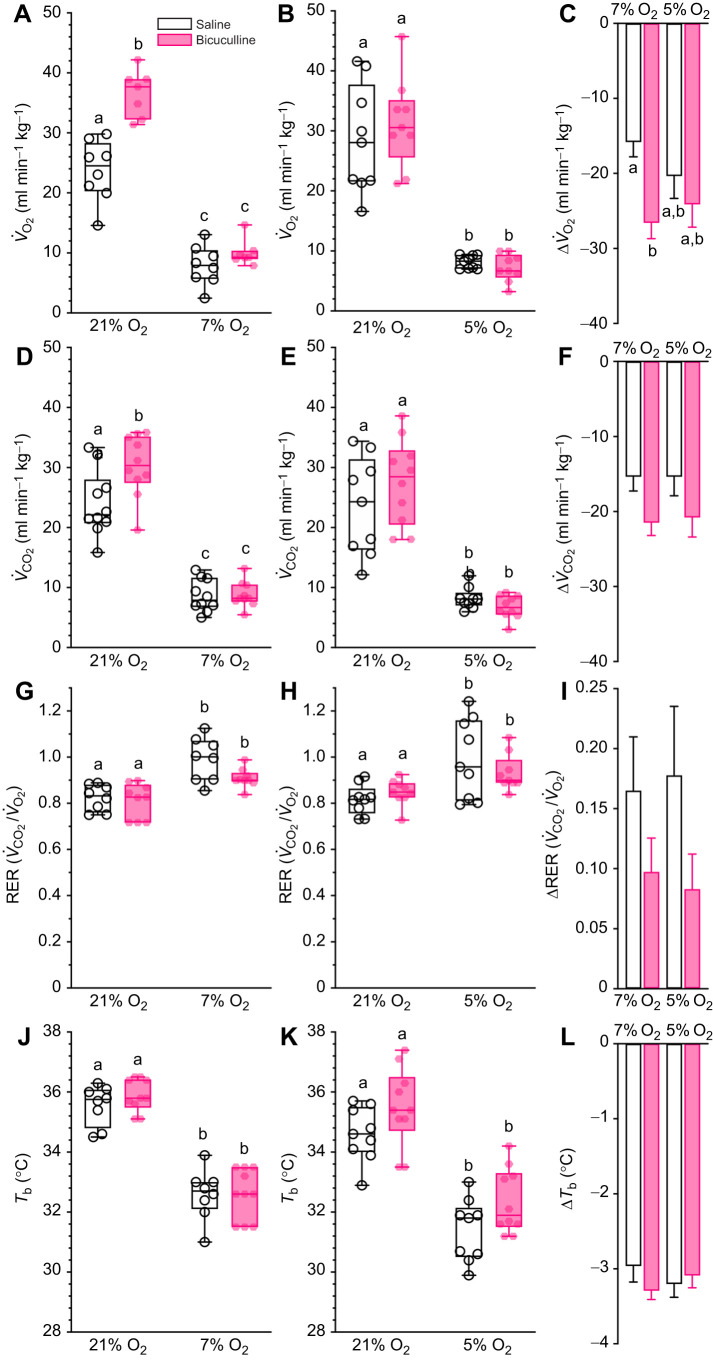
**Damaraland mole-rat metabolism and thermoregulation are minimally regulated by GABAergic signalling.** (A,B,D,E,G,H,J,K) Summaries of the rates of O_2_ consumption (*V̇*_O_2__; A,B), the rates of CO_2_ production (*V̇*_CO_2__; C,D), respiratory exchange ratios (RERs; G,H) and body temperatures (*T*_b_; J,K) from Damaraland mole-rats exposed to 21% O_2_, before and after intraperitoneal injections of saline alone (white bars and open symbols), or the γ-amino butyric acid type A (GABA_A_) receptor antagonist bicuculline (1 mg kg^−1^; dissolved in saline; pink bars and closed symbols), and subsequent exposure to acute hypoxia (7 or 5% O_2_; 1 h). Data are presented as box and whisker plots of min–max with individual replicates shown from *n=*7–10 biological replicates per dataset. (C,F,I,L) Summaries of the hypoxia-mediated change in *V̇*_O_2__ (C), *V̇*_CO_2__ (E), RER (I) and *T*_b_ (L) from animals treated as in A and B. Summary data are means±s.e.m. Different letters indicate significance as determined using a two-way ANOVA or mixed-effects model with Tukey's *post hoc* tests, *P*<0.05.

First, we evaluated the effect of bicuculline on *V̇*_O_2__ and *V̇*_CO_2__ as indicators of metabolic rate (Fig. 1A–F). Bicuculline had mixed effects on these variables in normoxia, but no effect in hypoxia. Specifically, both variables increased following drug injection during normoxia in protocol 1 (*F*_1,13_=31.04, *P*<0.0001 and *F*_1,13_=5.655, *P*=0.0034 for *V̇*_O_2__ and *V̇*_CO_2__, respectively; Fig. 1A,D), but not in protocol 2 (*F*_1,16_=0.2459, *P*=0.6267 and *F*_1,17_=0.5099, *P*=0.2389 for *V̇*_O_2__ and *V̇*_CO_2__, respectively; Fig. 1B,E), despite these experiments being conducted in random order and in the same animals. However, bicuculline-mediated changes during normoxia in protocol 1 were primarily due to lower average metabolic rates and reduced interindividual variability during normoxia in saline-treated animals in this group, which were not replicated in saline-treated animals in protocol 2. Similarly, bicuculline injection had a minimal effect on the HMR, with the changes in *V̇*_O_2__ and *V̇*_CO_2__ being largely similar between saline and drug treatment groups, except for the comparison between saline and drug in protocol 1 (*F*_1,29_=7.281, *P*=0.0115 and *F*_1,35_=0.02522, *P*=0.8947 for Δ*V̇*_O_2__ and Δ*V̇*_CO_2__, respectively; Fig. 1C,F).

We also calculated RERs to indirectly measure metabolic fuel substrate use (Fig. 1G–I). The RER was tightly maintained in normoxia, and following bicuculine injection, with the means from these experimental groups ranging from 0.8 to 0.85, indicating a mix of lipid and carbohydrate fuel use. The RER in bicuculline-treated animals breathing 5 or 7% O_2_ was not statistically different from measurements from saline-treated animals breathing hypoxic gas mixtures (*F*_1,15_=4.4944, *P*=0.0511 and *F*_1,16_=0.1594, *P*=0.6950 for 7 and 5% O_2_, respectively; Fig. 1G,H), and was similarly elevated relative to normoxic measurements in the same animals (*P*=0.0296 and 0.0146 for 7 and 5% O_2_, respectively). The magnitude of change of the RER was not affected by drug treatment (Fig. 1I).

Next, we measured *T*_b_ to gain insight into thermoregulation during hypoxia. We found that bicuculline injection had no effect on *T*_b_ during normoxia and did not alter the hypoxic decrease in *T*_b_ in either experimental protocol (*F*_1,16_=0.2870, *P*=0.5995 and *F*_1,17_=3.489, *P*=0.1728 for 7 and 5% O_2_, respectively; Fig. 1J,K). The magnitude of change of *T*_b_ was not affected by drug treatment (Fig. 1L).

### GABA_A_ receptor antagonism has opposing effects on breathing in normoxia versus hypoxia and abolishes the hypoxic change in the ACR

**Fig. 2.**
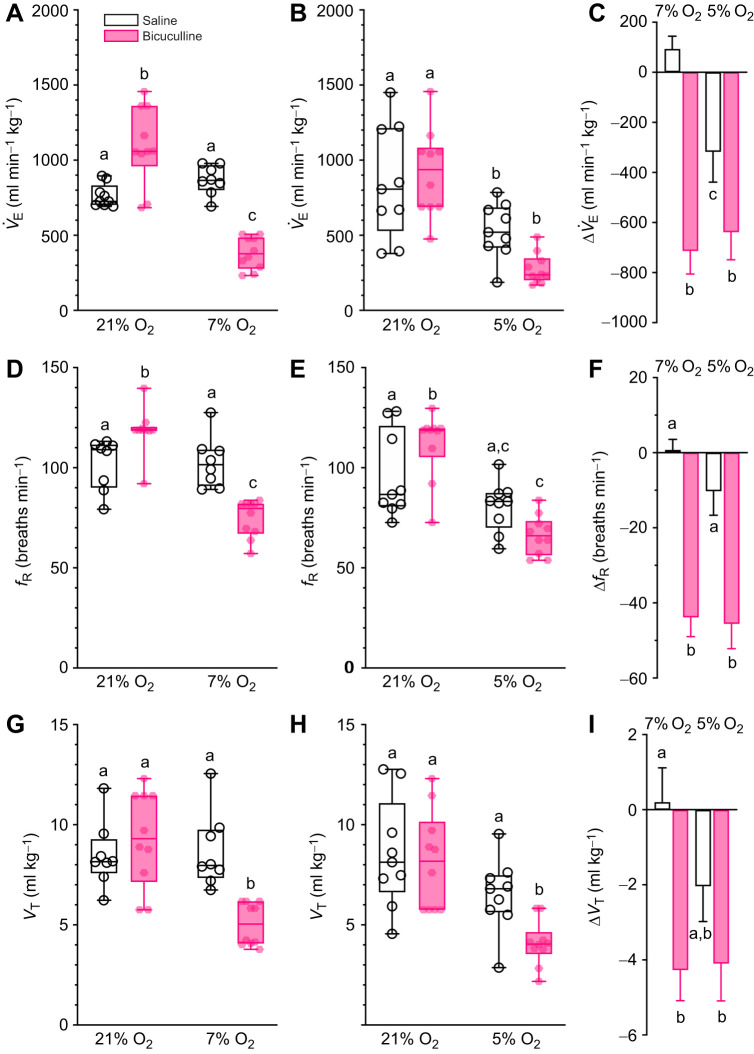
**GABA_A_ receptor antagonism regulates ventilation in hypoxia but not normoxia.** (A,B,D,E,G,H) Summaries of minute ventilation (*V̇*_E_; A,B), breathing frequency (*f*_R_; D,E) and tidal volume (*V*_T_; G,H) from Damaraland mole-rats exposed to 21% O_2_, before and after intraperitoneal injections of saline alone (white bars and open symbols), or the GABA_A_ receptor antagonist bicuculline (1 mg kg^−1^; dissolved in saline; pink bars and closed symbols), and subsequent exposure to acute hypoxia (7 or 5% O_2_; 1 h). Data are presented as box and whisker plots of min–max with individual replicates shown from *n=*7–10 biological replicates per dataset. (C,F,I) Summaries of the hypoxia-mediated change in *V̇*_E_ (C), *f*_R_ (E) and *V*_T_ from animals treated as in A and B. Summary data are means±s.e.m. Different letters indicate significance as determined using a two-way ANOVA or mixed-effects model with Tukey's *post hoc* tests, *P*<0.05.

To evaluate the role of GABA_A_ receptors in regulating Damaraland mole-rat breathing, we next measured changes in *V̇*_E_ and its component parameters (*f*_R_ and *V*_T_; Fig. 2). This analysis revealed that bicuculline had mixed effects on breathing in normoxia and more consistent effects in hypoxia. In normoxia, bicuculline increased baseline *V̇*_E_ and *f*_R_ in protocol 1 (*F*_1,16_=61.53, *P*<0.0001 and *F*_1,17_=3.896, *P*=0.8256 for *V̇*_E_ in 7 and 5% O_2_, respectively; and *F*_1,16_=51.02, *P*=0.009 and *F*_1,17_=9.239, *P*=0.0073 for *f*_R_ in 7 and 5% O_2_, respectively; Fig. 2A,B,D,E), but only *f*_R_ in protocol 2. Conversely, *V*_T_ was not affected by bicuculline in normoxia (*F*_1,16_=3.556, *P*=0.5625 and *F*_1,17_=4.116, *P*=0.9135 in 7 and 5% O_2_, respectively; Fig. 2G,H).

In protocol 1, bicuculline reduced *V̇*_E_ by ∼60% (*P*<0.0001; Fig. 2A). This change was driven by 60–70% reductions in both *f*_R_ and *V*_T_ (*P*<0.0001 for both; Fig. 2D,G). Conversely, in protocol 2, there was a trend in bicuculline-treated animals such that all ventilatory variables tended to decrease relative to saline-treated animals breathing the same hypoxic gas; however, this difference only reached significance for *V*_T_ (*P*=0.0349; Fig. 2G). Furthermore, in bicuculline-treated animals breathing 5% O_2_ (protocol 2), the HVR was enhanced such that *V̇*_E_, *f*_R_ and *V*_T_ were all decreased by ∼10–20% beyond the change observed in animals breathing 7% O_2_ in protocol 1.

As a result of these effects, bicuculline caused ventilatory changes to manifest in a shallower level of hypoxia, as evidenced by the appearance of a robust HVR in *V̇*_E_, *f*_R_ and *V*_T_ in animals treated with bicuculline and breathing 7% O_2_, which was not apparent in saline-treated animals breathing the same hypoxic gas (*F*_1,31_=27.71, *P*<0.0001, *F*_1,31_=50.53, *P*<0.001, and *F*_1,31_=12.48, *P*=0.0012, for *V̇*_E_, *f*_R_ and *V*_T_, respectively; Fig. 2C,F,I). In protocol 2, during which the natural HVR is apparent, bicuculline enhanced the HVR for both *V̇*_E_ and *f*_R_, but not *V*_T_ (*P*=0.0132, 0.006 and 0.3911, respectively).

**Fig. 3.**
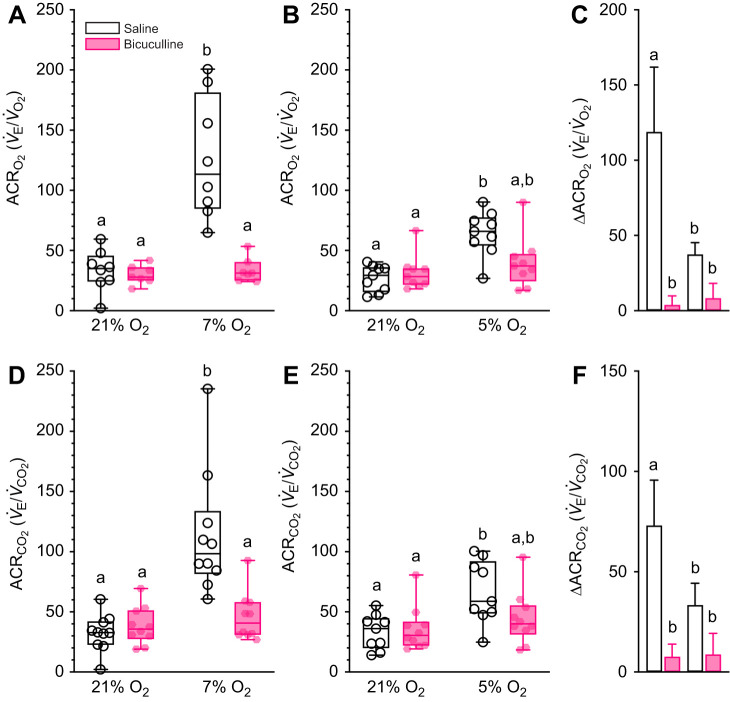
**GABA_A_ receptor antagonism prevents the hypoxic ventilatory response.** (A,B,D,E) Summaries of the air convection requirements of O_2_ (ACR_O_2__; A,B) and CO_2_ (ACR_CO_2__; D,E) from Damaraland mole-rats exposed to 21% O_2_, before and after intraperitoneal injections of saline alone (white bars and open symbols), or the GABA_A_ receptor antagonist bicuculline (1 mg kg^−1^; dissolved in saline; pink bars and closed symbols), and subsequent exposure to acute hypoxia (7 or 5% O_2_; 1 h). Data are presented as box and whiskers plots of min–max with individual replicates shown from *n=*7–10 biological replicates per dataset. (C,F) Summaries of the hypoxia-mediated change in ACR_O_2__ (C) and ACR_CO_2__ (F) from animals treated as in A and B. Summary data are means±s.e.m. Different letters indicate significance as determined using a 2-way ANOVA or mixed-effects model with Tukey's *post hoc* tests, *P*<0.05.

Finally, bicuculline treatment had no effect on either ACR in normoxia (*P*=0.8845 and >0.9999 for ACR_O_2__ and ACR_CO_2__, respectively, in protocol 1, and 0.8116 and 0.9586 for ACR_O_2__ and ACR_CO_2__, respectively, in protocol 2; Fig. 3A,B). Conversely, the inhibitory effect of bicuculline on breathing, combined with the natural HMR of this species, resulted in the abolishment of the hypoxia-driven change in ACR_O_2__ and ACR_CO_2__ in both experimental protocols (*F*_1,26_=21.36, *P*=0.0002 and *F*_1,26_=14.25, *P*=0.0026 for ACR_O_2__ and ACR_CO_2__, respectively, in protocol 1, and *F*_1,16_=13.43, *P*=0.0348 and *F*_1,16_=7.7, *P*=0.0129 for ACR_O_2__ and ACR_CO_2__, respectively, in protocol 2).

### Adenosine receptor antagonism increases metabolic rate in normoxia and reduces the hypoxia-mediated decreases in metabolic rate and thermogenesis

**Fig. 4.**
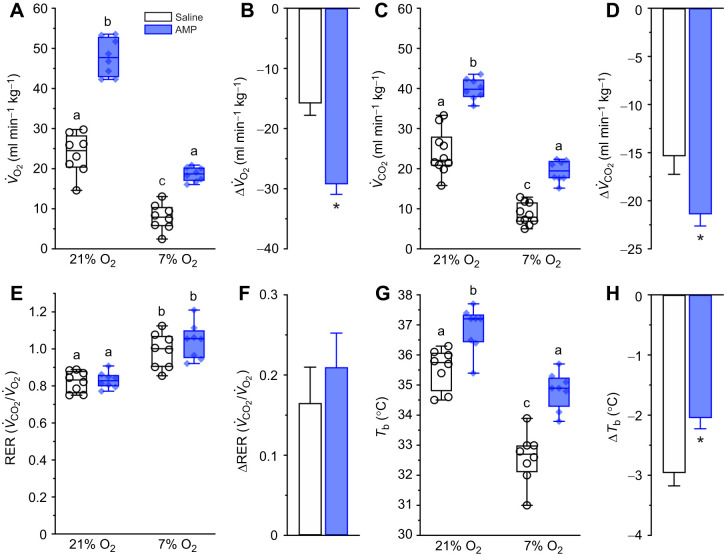
**Adenosine receptor antagonism reduces the hypoxic metabolic response and abolishes the hypoxia-mediated decrease in body temperature.** (A,C,E,G) Summaries of the rate of O_2_ consumption (*V̇*_O_2__; A), the rate of CO_2_ production (*V̇*_CO_2__; C), respiratory exchange ratios (RERs; E) and body temperatures (*T*_b_; G) from Damaraland mole-rats exposed to 21% O_2_, before and after injection of saline (white bars and open symbols) or the general adenosine receptor antagonist aminophylline (100 mg kg^−1^; dissolved in saline; blue bars and closed symbols), and subsequently exposed to acute hypoxia (7% O_2_ for 1 h). Data are presented as box and whisker plots of min–max with individual replicates shown from *n=*7–10 biological replicates per dataset. Different letters indicate significance as determined using a two-way ANOVA or mixed-effects model with Tukey's *post hoc* tests, *P<*0.05. (B,D,F,H) Summaries of the hypoxia-mediated change in *V̇*_O_2__ (B), *V̇*_CO_2__ (D), RER (F) and *T*_b_ (H) from animals treated as in A. Summary data are means±s.e.m. Asterisks indicate significant difference from saline controls as determined using paired Student’s *t*-tests, *P*<0.05.

Next, we evaluated the impact of aminophylline on *V̇*_O_2__ and *V̇*_CO_2__ (Fig. 4). Note that aminophylline treatment in 5% O_2_ was lethal to some animals and so we only completed the 7% O_2_ (protocol 1) dataset in this experimental group. Aminophylline had a significant effect on both variables (*F*_1,14_=26.43, *P*=0.0002 and *F*_1,14_=26.24, *P*=0.0002 for *V̇*_O_2__ and *V̇*_CO_2__, respectively). Specifically, inhibition of adenosine receptors increased both *V̇*_O_2__ and *V̇*_CO_2__ by ∼2-fold in normoxia relative to saline-treated animals (*P*<0.0001 for both variables). In aminophylline-treated animals breathing 7% O_2_, both *V̇*_O_2__ and *V̇*_CO_2__ returned to pre-drug baseline levels, as compared with the ∼55–65% hypoxia-mediated reductions in these variables in saline-treated animals (*P*=0.0022 and 0.0016 for *V̇*_O_2__ and *V̇*_CO_2__, respectively). Nonetheless, the magnitude of change of both *V̇*_O_2__ and *V̇*_CO_2__ following the onset of hypoxia was greater in aminophylline-treated animals than saline-treated animals because of the drug-mediated elevation in these variables in normoxia (Fig. 4B,D). Despite these changes in *V̇*_O_2__ and *V̇*_CO_2__ with aminophylline treatment, the ratio between these variables (i.e. the RER) was tightly maintained in both normoxia and hypoxia and was not significantly different from saline-treated animals in the same conditions (Fig. 4E). Similarly, the magnitude of the hypoxia-mediated change in the RER was not affected by aminophylline (Fig. 4F).

Aminophylline injection had similar and significant impacts on *T*_b_ (*F*_1,14_=13.19, *P*=0.0027; Fig. 4G,H). Specifically, aminophylline increased *T*_b_ by∼1.5°C in normoxia (*P*=0.0021). In hypoxia, the *T*_b_ of aminophylline-treated animals returned to pre-drug baseline levels. However, because of the drug-mediated increase in *T*_b_ in normoxia, there was still a net decrease in *T*_b_ following the transition to hypoxia in aminophylline-treated animals (Fig. 4H), although the magnitude of this hypoxic change was decreased relative to saline-treated animals.

### Aminophylline increases ventilation in normoxia and abolishes the relative hypoxic ventilatory response

**Fig. 5.**
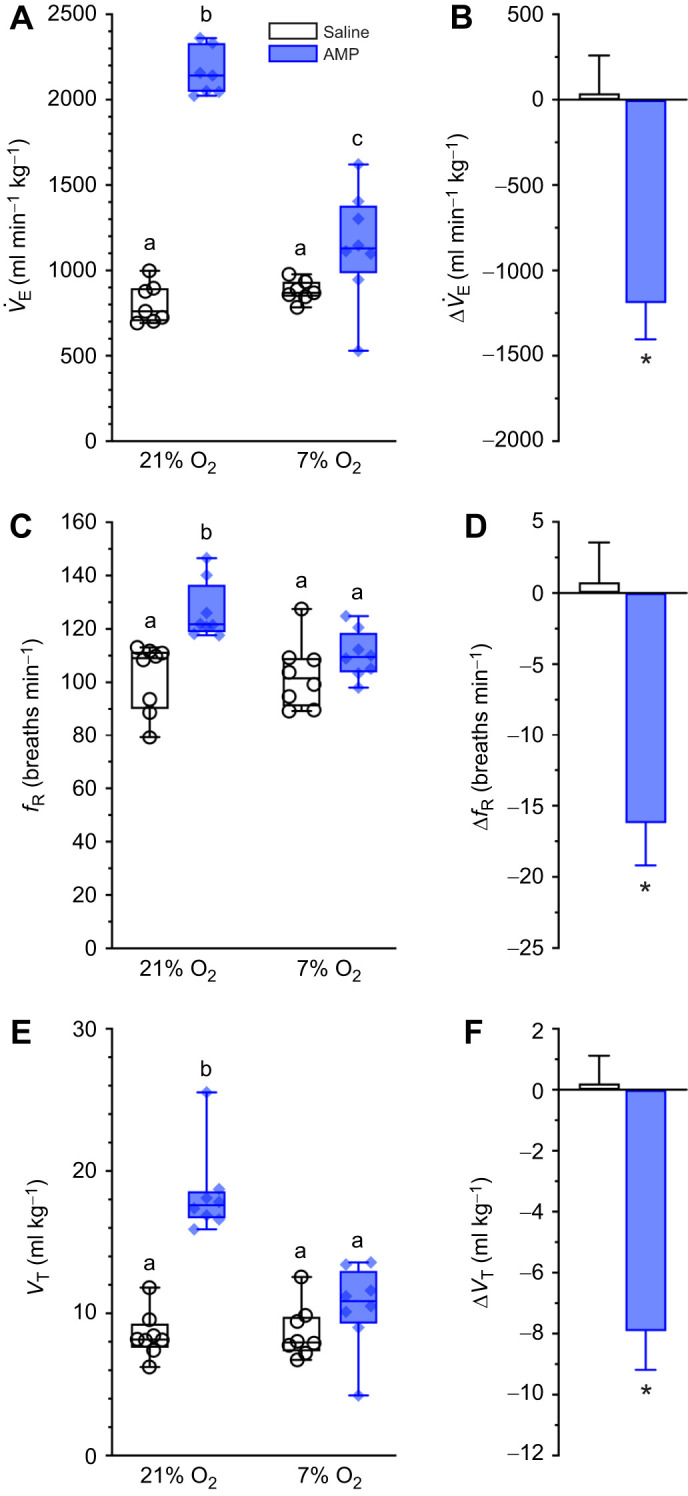
**Adenosine receptor antagonism induces hyperventilation in normoxia but not hypoxia.** (A,C,E) Summaries of minute ventilation (*V̇*_E_; A), breathing frequency (*f*_R_; C) and tidal volume (*V*_T_; E) from Damaraland mole-rats exposed to 21% O_2_, before and after injection of saline (white bars and open symbols) or aminophylline (100 mg kg^−1^; dissolved in saline; blue bars and closed symbols), and subsequently exposed to acute hypoxia (7% O_2_ for 1 h). Data are presented as box and whisker plots of min–max with individual replicates shown from *n=*7–10 biological replicates per dataset. Different letters indicate significance as determined using a two-way ANOVA or mixed-effects model with Tukey's *post hoc* tests, *P<*0.05. (B,D,F) Summaries of the hypoxia-mediated change in *V̇*_E_ (B), *f*_R_ (D) and *V*_T_ (F) from animals treated as in A. Summary data are means±s.e.m. Asterisks indicate significant difference from saline controls as determined using paired Student’s *t*-tests, *P<*0.05.

Aminophylline markedly increases all ventilatory variables in normoxia, with *V̇*_E_ and *V*_T_ each increasing 2- to 3-fold and *f*_R_ increasing mildly (*F*_1,14_=34.37, *P*<0.0001 for *V̇*_E_, *F*_1,14_=7.74, *P*=0.0004 for *f*_R_, and *F*_1,14_=45.14, *P*<0.0001 for *V*_T_; Fig. 5). These changes were largely reversed during the subsequent hypoxia exposure (*P*=0.7266, 0.3967 and 0.3202 versus normoxia, respectively). As with metabolism, the magnitude of change in all ventilatory parameters was greater in aminophylline-treated animals than in animals treated with saline, but this was due to the large increase in ventilation that occurs with drug treatment in normoxia, and which is subsequently reversed during hypoxia.

**Fig. 6.**
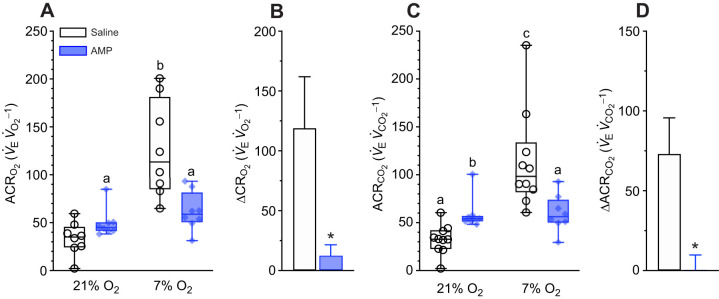
**Adenosine receptor antagonism prevents the relative hypoxic ventilatory response.** (A,C) Summaries of the air convection requirements of O_2_ (ACR_O_2__; A) and CO_2_ (ACR_CO_2__; C) from Damaraland mole-rats exposed to 21% O_2_, before and after injection of saline (white bars and open symbols) or aminophylline (100 mg kg^−1^; dissolved in saline; blue bars and closed symbols), and subsequently exposed to acute hypoxia (7% O_2_ for 1 h). Data are presented as box and whisker plots of min–max with individual replicates shown from *n=*7–10 biological replicates per dataset. Different letters indicate significance as determined using a two-way ANOVA or mixed-effects model with Tukey's *post hoc* tests, *P<*0.05. (B,D) Summaries of the hypoxia-mediated change in ACR_O_2__ (B) and ACR_CO_2__ (D) from animals treated as in A and C. Summary data are means±s.e.m. Asterisks indicate significant difference from saline controls as determined using paired Student’s *t*-tests, *P<*0.05.

Importantly, we did not observe a hypoxic ventilatory response in 7% O_2_ in our control experiments because this response is blunted in Damaraland mole-rats and does not manifest until ∼3–5% O_2_ (Ivy et al., 2020). However, as this severe level of hypoxia was lethal when applied concomitantly with aminophylline (data not shown), we could not directly test the role of adenosine on the hypoxia-mediated changes in ventilation in this species at or below 5% O_2_. Therefore, we instead relied upon ACRs to determine whether adenosine receptors mediate the HVR in Damaraland mole-rats in 7% O_2_ because these variables incorporate metabolic changes along with the HVR. Thus, the robust HMR of this species drives a relative HVR in 7% O_2_ despite the lack of overt change in any ventilatory variable. Aminophylline treatment had no impact on the ACRs in normoxia (*P*=0.4626 and 0.2409 for ACR_O_2__ and ACR_CO_2__, respectively; Fig. 6), indicating that the increases in metabolism, thermogenesis and ventilation following inhibition of adenosinergic signalling are coupled and balanced and that increased metabolic demand is well matched by increases in O_2_ delivery in normoxia. Conversely, aminophylline abolished the hypoxia-mediated increases in ACR_O_2__ (*P*=0.0641 and 0.6438 versus pre- and post-injection values in the same animals; Fig. 6B) and ACR_CO_2__ (*P*=0.2232 and >0.999 versus pre- and post-injection values in the same animals; Fig. 6D).

## DISCUSSION

In the present study, we evaluated a role for GABAergic and adenosinergic signalling in regulating the hypoxic ventilatory, metabolic and thermoregulatory responses of fossorial Damaraland mole-rats to acute hypoxia. Our study yielded several important findings. First, adenosine appears to be the primary neurotransmitter regulating metabolism and thermogenesis of Damaraland mole-rats because aminophylline treatment substantially increases metabolic rate and *T*_b_ in both normoxia and hypoxia and reduces hypoxia-mediated decreases in both variables. As a result, aminophylline abolishes the relative HVR (i.e. the ACR) through interactions with metabolic but not ventilatory parameters. Conversely, GABA receptors play a lesser role in regulating metabolism or thermoregulation in normoxia or hypoxia but have significant and opposing effects on ventilation between normoxic and hypoxic treatments. Specifically, bicuculline increases breathing in normoxia but decreases breathing in hypoxia, resulting in a more sensitive HVR in these conditions. However, the net effect of bicuculline on the ACR is to prevent hypoxia-mediated changes in this variable, and thus bicuculline abolishes the relative HVR. This is intriguing and suggests that GABA plays an inhibitory role in regulating breathing in normoxia but an excitatory role in hypoxia.

### Adenosinergic signalling mediates metabolism and thermogenesis

We report that Damaraland mole-rats have a robust HMR to acute hypoxia, which is partially mediated by decreased thermoregulation. These findings are also generally consistent with responses to hypoxia in numerous other small fossorial mammal species (Arieli and Ar, 1979; Arieli et al., 1977; Barros et al., 2004; Boggs et al., 1998; Frappell et al., 1992, 1994; Guppy and Withers, 1999; Tomasco et al., 2010), including several related species of African mole-rats (Devereaux and Pamenter, 2020; Ivy et al., 2020; Pamenter, 2022). Our data extends these previous findings to show that the HMR and hypoxic decrease in thermogenesis in Damaraland mole-rats are partially mediated by adenosinergic signalling. This finding differs somewhat from a previous study (Pamenter et al., 2015) in naked mole-rats, in which the hypoxic decrease in *V̇*_O_2__ was not impacted by aminophylline (although the hypoxic decrease in *V̇*_CO_2__ was halved by this drug).

In other small mammals, including both hypoxia-tolerant and -intolerant fossorial species, adenosine receptors initiate and maintain the HMR and decreases in *T*_b_ during entry into torpor. For example, activating adenosine receptors reduces metabolic rate and thermogenesis, and induces torpor in arctic ground squirrels and rats (Jinka et al., 2011; Tupone et al., 2013). In contrast, blocking adenosine receptors prevents or reverses these changes during intra-day or fasting-induced torpor in rats (Tupone et al., 2013) and mice (Iliff and Swoap, 2010, 2012; Swoap et al., 2012), and in hibernating arctic ground squirrels (Jinka et al., 2011; Olson et al., 2013).

Conversely, we demonstrated that metabolic and thermoregulatory responses to hypoxia are likely not mediated by GABAergic signalling in Damaraland mole-rats. An important caveat is that the hypoxic change in *V̇*_O_2__ is enhanced by bicuculine in protocol 1; however, this is not the case for protocol 2, and bicuculine (unlike aminophylline) does not alter the hypoxic value for *V̇*_O_2__ relative to saline treatment in either protocol. Thus, the preponderance of evidence suggests a minimal role for GABAergic signalling in mediating the HMR. This finding agrees with a previous study in naked mole-rats, in which the hypoxic decrease in *V̇*_O_2__ was not affected by bicuculline (Dzal et al., 2019).

### GABAergic signalling modulates the HVR

We demonstrate that Damaraland mole-rats have a blunted HVR that does not manifest until 5% O_2_, which is consistent with a previous study in this species (Ivy et al., 2020), and with other fossorial species, in which the HVR is typically activated in the range of 5–12.5% O_2_ (Ivy et al., 2020; Tomasco et al., 2010). Blocking GABA_A_ receptors in normoxia increases ventilatory parameters, suggesting that there is an inhibitory drag on ventilation from active GABAergic signalling in normoxia. Remarkably, our results suggest that this drag on ventilation is reversed in hypoxia and that GABAergic signalling, at least within the ventilatory control circuits, is net excitatory when O_2_ is limited. As a result, GABA_A_ receptors play a role in dampening ventilation during acute hypoxia in Damaraland mole-rats and reduce the sensitivity of ventilation to hypoxia, such that blocking GABA_A_ receptors causes the HVR to manifest at 7% instead of 5% O_2_ (which is the HVR threshold in saline-treated animals). Notably, however, this role is different from that in naked mole-rats, in which bicuculline prevents the hypoxic decrease in ventilation. This suggests that GABA may act at different points in the ventilatory control system and may directly modulate other inhibitory ventilatory inputs than in naked mole-rats.

The apparent reversal of GABAergic signalling is fascinating and suggests one of two possibilities: either (1) the Cl^−^ reversal potential (and thus the reversal potential of the GABA_A_ receptors: *E*_GABA_) shifts during hypoxia in this species, rendering GABAergic signalling mildly excitatory in hypoxia, or (2) hypoxia directly activates inhibitory interneurons that synapse onto and subsequently inhibit excitatory ventilatory inputs, thereby reducing the ventilatory drive in hypoxia. Given that bicuculline increased the sensitivity of breathing to hypoxia, the former explanation is more likely.

Although *E*_GABA_ is almost always hyperpolarizing relative to neuronal membrane potential in adult hypoxia-intolerant mammals, there are exceptions. For example, in isolated rat carotid body petrosal neurons, *E*_GABA_ is depolarizing relative to resting membrane potential (Zhang et al., 2009). In addition, there are several examples in the nervous systems of other hypoxia- and anoxia-tolerant species in which *E*_GABA_ is depolarizing (and thus initially excitatory) and/or shifts from hyperpolarizing to depolarizing with changing environments. For example, in anoxia-tolerant pond snails (*Lymnaea stagnalis*), *E*_GABA_ shifts between an inhibitory and excitatory phenotype depending on the environmental photoperiod (Buck et al., 2017), presumably to help this animal overwinter in anoxic waters. Similarly, *E*_GABA_ is depolarized relative to resting membrane potential in the cortex of anoxia-tolerant freshwater turtles (*Chrysemys picta bellii*), and robust GABAergic activation in anoxia or ischemia causes neurons to mildly depolarize (Buck et al., 2012; Hogg et al., 2015; Pamenter et al., 2011). A similar phenotype has also been reported in naked mole-rats, in which *E*_GABA_ is depolarized by ∼10 mV relative to the neuronal membrane potential, which is like the difference between *E*_GABA_ and membrane potential in turtle neurons (Zions et al., 2020). Unfortunately, there is no information available regarding *E*_GABA_ in hypoxic naked mole-rat brain or in Damaraland mole-rats in any condition, but our results suggest that *E*_GABA_ is also excitatory in normoxia in Damaraland mole-rats and may shift to an inhibitory phenotype in hypoxia. Further experiments are warranted to evaluate *E*_GABA_ in both naked and Damaraland mole-rats in hypoxia.

In contrast, the role of adenosine receptors in mediating ventilation and the HVR in Damaraland mole-rats is less clear; aminophylline increases *V̇*_E_ in both normoxia and hypoxia but also abolishes the hypoxia-mediated increase of the ACRs. In general, this finding is consistent with other mammals, in which adenosine is known to accumulate in the blood during hypoxia and modulate the HVR (Drumm et al., 2004). For example, aminophylline reduces (but does not abolish) the second, declining component of the biphasic HVR in awake humans (Easton and Anthonisen, 1988), cats (Long and Anthonisen, 1994), rats (Maskrey and Westwood, 1994) and lambs (Koos et al., 2005), and in anaesthetized piglets (Darnall, 1985). Furthermore, in studies in which the effect of aminophylline on ventilation is examined along with its effects on metabolism, aminophylline always inhibits the HVR in addition to the HMR (Barros et al., 2006; Koos et al., 2005; Maxwell et al., 1986). Therefore, it is likely that adenosine receptor inhibition would affect hypoxic ventilation in Damaraland mole-rats if it were ethically possible (i.e. not lethal) to expose them to lower levels of O_2_ in combination with aminophylline treatment.

### Study limitations

Bicuculline and aminophylline have clear impacts on breathing and metabolic variables in both normoxia and hypoxia, which demonstrates that these drugs, and at the concentrations we employ in our study, are efficacious in this species. Nonetheless, drugs were injected intraperitoneally and thus their specificity of action must be interpreted with caution. However, intraperitoneal injections of these pharmacological agents have concentration decay and time profiles similar to those of intravenous injections (Asheim et al., 2008), and intraperitoneal injections of both drugs used in the present study have been used previously in studies in other mammals to evaluate similar physiological and behavioural responses (e.g. Chung et al., 2016; Crisanti and Fewell, 1999; Pamenter et al., 2015; Zimmerman et al., 2003). Furthermore, aminophylline and bicuculline both cross the blood–brain barrier and can thus interact with adenosine receptors within the central nervous system (Remler and Marcussen, 1985; Veng-Pedersen et al., 1983). However, GABA_A_ and adenosine receptors are also found outside of the central nervous system and thus our results only allow us to conclude that the effects we observed are mediated by these receptors, but not which population of receptors are involved in this regulation. It is also important to note that whereas bicuculline is primarily specific for GABA_A_ receptors (Johnston, 2013), aminophylline is less specific. Indeed, aminophylline has many off-target effects at the cellular level, including as a weak inhibitor of some transcriptional pathways and phosphodiesterase, as well as varied impacts on inflammatory pathways and inflammatory cell survival, among others (Barnes, 2010). However, these effects are generally weak or minimal. For example, at the highest concentration of aminophylline tested, phosphodiesterase activity is inhibited by ∼14–20% (Polson et al., 1978). Furthermore, most of these off-target effects would not manifest at the physiological level within the 1–2 h period of our experiments, which should limit any confounding impact on our data.

### Conclusions

In the present study, we demonstrated that GABA_A_ and adenosine receptors contribute to the hypoxic metabolic and ventilatory responses in Damaraland mole-rats. This mechanistic phenotype is unlike that of most non-fossorial mammals studied to date, which instead rely on glutamatergic signalling to mediate the HVR (Pamenter and Powell, 2016). Regarding adenosine, our findings differ from previous studies in closely related naked mole-rats, in which adenosinergic signalling is central to the manifestation of the HVR and plays a lesser role in mediating the HMR. However, our findings are generally consistent with studies in other small rodents in which adenosine is a key mediator of hypometabolism during torpor bouts and hibernation. The mechanism via which adenosine receptors modulate metabolism has not been elucidated in any species and warrants further investigation. Further studies in other fossorial mammals are also warranted to determine whether GABAergic signalling is the predominant neurotransmission pathway regulating ventilation and the HVR in other fossorial species. In addition, research is required to understand the mechanism via which GABA receptors modulate ventilation in such species.

## References

[JEB246186C1] Anyan, J. J., Seney, M. L., Holley, A., Bengston, L., Goldman, B. D., Forger, N. G. and Holmes, M. M. (2011). Social status and sex effects on neural morphology in Damaraland mole-rats, *Fukomys damarensis*. *Brain Behav. Evol.* 77, 291-298. 10.1159/000328640 21701152 PMC3182041

[JEB246186C2] Arieli, R. and Ar, A. (1979). Ventilation of a fossorial mammal (*Spalax ehrenbergi*) in hypoxic and hypercapnic conditions. *J. Appl. Physiol. Respir. Environ. Exerc. Physiol.* 47, 1011-1017. 10.1152/jappl.1979.47.5.1011 511701

[JEB246186C3] Arieli, R., Ar, A. and Shkolnik, A. (1977). Metabolic responses of a fossorial rodent (*Spalax ehrenbergi*) to simulated burrow conditions. *Physiol. Zool.* 50, 61-75. 10.1086/physzool.50.1.30155716

[JEB246186C4] Asheim, P., Spigset, O., Aasarod, K., Walstad, R. A., Uggen, P. E., Zahlsen, K. and Aadahl, P. (2008). Pharmacokinetics of intraperitoneally instilled aminophylline, terbutaline and tobramycin in pigs. *Acta Anaesthesiol. Scand.* 52, 243-248. 10.1111/j.1399-6576.2007.01535.x 18005375

[JEB246186C5] Barnes, P. J. (2010). Theophylline. *Pharmaceuticals* 3, 725-747. 10.3390/ph3030725 27713276 PMC4033977

[JEB246186C6] Barros, R. C. H., Abe, A. S., Carnio, E. C. and Branco, L. G. S. (2004). Regulation of breathing and body temperature of a burrowing rodent during hypoxic-hypercapnia. *Comp. Biochem. Physiol. A Mol. Integr. Physiol.* 138, 97-104. 10.1016/j.cbpb.2004.03.011 15165576

[JEB246186C7] Barros, R. C., Branco, L. G. and Carnio, E. C. (2006). Respiratory and body temperature modulation by adenosine A1 receptors in the anteroventral preoptic region during normoxia and hypoxia. *Respir. Physiol. Neurobiol.* 153, 115-125. 10.1016/j.resp.2005.09.013 16352472

[JEB246186C8] Beart, P. M. and Johnston, G. A. (1972). Bicuculline and GABA-metabolising enzymes. *Brain Res.* 38, 226-227. 10.1016/0006-8993(72)90609-9 4552595

[JEB246186C9] Boggs, D. F., Frappell, P. B. and Kilgore, D. L. Jr (1998). Ventilatory, cardiovascular and metabolic responses to hypoxia and hypercapnia in the armadillo. *Respir. Physiol.* 113, 101-109. 10.1016/S0034-5687(98)00046-2 9832229

[JEB246186C10] Buck, L. T. (2004). Adenosine as a signal for ion channel arrest in anoxia-tolerant organisms. *Comp. Biochem. Physiol. B Biochem. Mol. Biol.* 139, 401-414. 10.1016/j.cbpc.2004.04.002 15544964

[JEB246186C11] Buck, L. T., Hogg, D. W., Rodgers-Garlick, C. and Pamenter, M. E. (2012). The painted turtle as a model of natural anoxia tolerance: role of the neurotransmitter GABA. In: *Turtles: Anatomy, Ecology and Conservation* (ed. S. R. MJ Cosgrove). New York: Nova Science.

[JEB246186C12] Buck, L. T., Bond, H. C. and Malik, A. (2017). Assessment of anoxia tolerance and photoperiod dependence of GABAergic polarity in the pond snail Lymnaea stagnalis. *Comp. Biochem. Physiol. A Mol. Integr. Physiol.* 203, 193-200. 10.1016/j.cbpa.2016.09.016 27664385

[JEB246186C13] Buffenstein, R., Amoroso, V., Andziak, B., Avdieiev, S., Azpurua, J., Barker, A. J., Bennett, N. C., Brieno-Enriquez, M. A., Bronner, G. N., Coen, C. et al. (2022). The naked truth: a comprehensive clarification and classification of current ‘myths’ in naked mole-rat biology. *Biol. Rev. Camb. Philos. Soc.* 97, 115-140. 10.1111/brv.12791 34476892 PMC9277573

[JEB246186C14] Cheng, H., Sebaa, R., Malholtra, N., Lacoste, B., El Hankouri, Z., Kirby, A., Bennett, N. C., Van Jaarsveld, B., Hart, D. W., Tattersall, G. J. et al. (2021). Naked mole-rat brown fat thermogenesis is diminished during hypoxia through a rapid decrease in UCP1. *Nat. Commun.* 12, 6801. 10.1038/s41467-021-27170-2 34815412 PMC8610999

[JEB246186C15] Chung, D., Dzal, Y. A., Seow, A., Milsom, W. K. and Pamenter, M. E. (2016). Naked mole rats exhibit metabolic but not ventilatory plasticity following chronic sustained hypoxia. *Proc. Biol. Sci.* 283, 20160216. 10.1098/rspb.2016.0216 27009224 PMC4822471

[JEB246186C16] Clayson, M. S., Devereaux, M. E. M. and Pamenter, M. E. (2020). Neurokinin-1 receptor activation is sufficient to restore the hypercapnic ventilatory response in the Substance P-deficient naked mole-rat. *Am. J. Physiol. Regul. Integr. Comp. Physiol.* 318, R712-R721. 10.1152/ajpregu.00251.2019 31967860 PMC7191416

[JEB246186C17] Crisanti, K. C. and Fewell, J. E. (1999). Aminophylline alters the core temperature response to acute hypoxemia in newborn and older guinea pigs. *Am. J. Physiol.* 277, R829-R835. 10.1152/ajpregu.1999.277.3.R829 10484500

[JEB246186C18] Curtis, D. R., Duggan, A. W., Felix, D. and Johnston, G. A. (1970a). Bicuculline and central GABA receptors. *Nature* 228, 676-677. 10.1038/228676a0 5474943

[JEB246186C19] Curtis, D. R., Duggan, A. W., Felix, D. and Johnston, G. A. (1970b). GABA, bicuculline and central inhibition. *Nature* 226, 1222-1224. 10.1038/2261222a0 4393081

[JEB246186C20] Darnall, R. A. Jr (1985). Aminophylline reduces hypoxic ventilatory depression: possible role of adenosine. *Pediatr. Res.* 19, 706-710. 10.1203/00006450-198507000-00014 3927252

[JEB246186C21] Devereaux, M. E. M. and Pamenter, M. E. (2020). Fossorial giant Zambian mole-rats have blunted ventilatory responses to environmental hypoxia and hypercapnia. *Comp. Biochem. Physiol. A Mol. Integr. Physiol.* 243, 110672. 10.1016/j.cbpa.2020.110672 32032753

[JEB246186C22] Devereaux, M. E. M., Chiasson, S., Brennan, K. F. and Pamenter, M. E. (2023). The glutamatergic drive to breathe is reduced in severe but not moderate hypoxia in Damaraland mole-rats. *J. Exp. Biol.* 226, jeb246185. 10.1242/jeb.246185 PMC10565110 37589556

[JEB246186C23] Drorbaugh, J. E. and Fenn, W. O. (1955). A barometric method for measuring ventilation in newborn infants. *Pediatrics* 16, 81-87. 10.1542/peds.16.1.81 14394741

[JEB246186C24] Drumm, D., Hoefer, M., Juhasz, J., Huszar, E. and Sybrecht, G. W. (2004). Plasma adenosine during investigation of hypoxic ventilatory response. *Sleep Breath* 8, 31-41. 10.1007/s11325-004-0031-5 15026936

[JEB246186C25] Dzal, Y. A., Seow, A., Borecky, L. G., Chung, D., Gill, S. K. G., Milsom, W. K. and Pamenter, M. E. (2019). Glutamatergic receptors modulate normoxic but not hypoxic ventilation and metabolism in naked mole rats. *Front. Physiol.* 10, 106. 10.3389/fphys.2019.00106 30833905 PMC6387965

[JEB246186C26] Easton, P. A. and Anthonisen, N. R. (1988). Ventilatory response to sustained hypoxia after pretreatment with aminophylline. *J. Appl. Physiol.* 64, 1445-1450. 10.1152/jappl.1988.64.4.1445 3378980

[JEB246186C27] Frappell, P., Lanthier, C., Baudinette, R. V. and Mortola, J. P. (1992). Metabolism and ventilation in acute hypoxia: a comparative analysis in small mammalian species. *Am. J. Physiol.* 262, R1040-R1046. 10.1152/ajpregu.1992.262.6.R1040 1621857

[JEB246186C28] Frappell, P. B., Franklin, C. E. and Grigg, G. C. (1994). Ventilatory and metabolic responses to hypoxia in the echidna *Tachyglossus aculeatus*. *Am. J. Physiol. Regul. Integr. Comp. Physiol.* 267, R1510-R1515. 10.1152/ajpregu.1994.267.6.R1510 7810760

[JEB246186C29] Gale, K. and Casu, M. (1981). Dynamic utilization of GABA in substantia nigra: regulation by dopamine and GABA in the striatum, and its clinical and behavioral implications. *Mol. Cell. Biochem.* 39, 369-405. 10.1007/BF00232586 6118827

[JEB246186C30] Guppy, M. and Withers, P. (1999). Metabolic depression in animals: physiological perspectives and biochemical generalizations. *Biol. Rev. Camb. Philos. Soc.* 74, 1-40. 10.1017/S0006323198005258 10396183

[JEB246186C31] Hogg, D. W., Pamenter, M. E., Dukoff, D. J. and Buck, L. T. (2015). Decreases in mitochondrial reactive oxygen species initiate GABA_A_ receptor-mediated electrical suppression in anoxia-tolerant turtle neurons. *J. Physiol.* 593, 2311-2326. 10.1113/JP270474 25781154 PMC4457194

[JEB246186C32] Iliff, B. W. and Swoap, S. J. (2010). Treatment with an adenosine receptor antagonist completely blocks fasting-induced torpor in mice. *FASEB J.* 24, 992.2.

[JEB246186C33] Iliff, B. W. and Swoap, S. J. (2012). Central adenosine receptor signaling is necessary for daily torpor in mice. *Am. J. Physiol. Regul. Integr. Comp. Physiol.* 303, R477-R484. 10.1152/ajpregu.00081.2012 22785425

[JEB246186C34] Ivy, C. M., Sprenger, R. J., Bennett, N. C., Van Jaarsveld, B., Hart, D. W., Kirby, A. M., Yaghoubi, D., Storey, K. B., Milsom, W. K. and Pamenter, M. E. (2020). The hypoxia tolerance of eight related African mole-rat species rivals that of naked mole-rats, despite divergent ventilatory and metabolic strategies in severe hypoxia. *Acta Physiologica* 228, e13436. 10.1111/apha.13436 31885213

[JEB246186C35] Jinka, T. R., Toien, O. and Drew, K. L. (2011). Season primes the brain in an arctic hibernator to facilitate entrance into torpor mediated by adenosine A_1_ receptors. *J. Neurosci.* 31, 10752-10758. 10.1523/JNEUROSCI.1240-11.2011 21795527 PMC3325781

[JEB246186C36] Johnston, G. A. (2013). Advantages of an antagonist: bicuculline and other GABA antagonists. *Br. J. Pharmacol.* 169, 328-336. 10.1111/bph.12127 23425285 PMC3651659

[JEB246186C37] Kirby, A. M., Fairman, G. D. and Pamenter, M. E. (2018). Atypical behavioural, metabolic and thermoregulatory responses to hypoxia in the naked mole rat (*Heterocephalus glaber*). *J. Zool.* 305, 106-115. 10.1111/jzo.12542

[JEB246186C38] Koos, B. J., Kawasaki, Y., Kim, Y. H. and Bohorquez, F. (2005). Adenosine A2A-receptor blockade abolishes the roll-off respiratory response to hypoxia in awake lambs. *Am. J. Physiol. Regul. Integr. Comp. Physiol.* 288, R1185-R1194. 10.1152/ajpregu.00723.2004 15618344

[JEB246186C39] Lighton, J. (2008). *Measuring Metabolic Rates: A Manual for Scientists*. Oxford: Oxford University Press.

[JEB246186C40] Lohmann, S. M. and Miech, R. P. (1976). Theophylline metabolism by the rat liver microsomal system. *J. Pharmacol. Exp. Ther.* 196, 213-225. 1246012

[JEB246186C41] Long, W. Q. and Anthonisen, N. R. (1994). Aminophylline partially blocks ventilatory depression with hypoxia in the awake cat. *Can. J. Physiol. Pharmacol.* 72, 673-678. 10.1139/y94-095 7954099

[JEB246186C42] Martin, R. H. (1946). Pharmacological action of aminophylline. *Univ. West. Ont. Med. J.* 16, 118-125. 20997536

[JEB246186C43] Maskrey, M. and Westwood, K. J. (1994). Dual effect of aminophylline on the ventilatory response to hypoxia in the rat. *J. Basic Clin. Physiol. Pharmacol.* 5, 227-238. 10.1515/JBCPP.1994.5.3-4.227 8736033

[JEB246186C44] Maxwell, D. L., Fuller, R. W., Nolop, K. B., Dixon, C. M. and Hughes, J. M. (1986). Effects of adenosine on ventilatory responses to hypoxia and hypercapnia in humans. *J. Appl. Physiol.* 61, 1762-1766. 10.1152/jappl.1986.61.5.1762 3781985

[JEB246186C45] Mortola, J. P., Rezzonico, R. and Lanthier, C. (1989). Ventilation and oxygen consumption during acute hypoxia in newborn mammals: a comparative analysis. *Respir. Physiol.* 78, 31-43. 10.1016/0034-5687(89)90140-0 2813986

[JEB246186C46] Olson, J. M., Jinka, T. R., Larson, L. K., Danielson, J. J., Moore, J. T., Carpluck, J. and Drew, K. L. (2013). Circannual rhythm in body temperature, torpor, and sensitivity to A_1_ adenosine receptor agonist in arctic ground squirrels. *J. Biol. Rhythms* 28, 201-207. 10.1177/0748730413490667 23735499 PMC4423736

[JEB246186C47] Pamenter, M. E. (2022). Adaptations to a hypoxic lifestyle in naked mole-rats. *J. Exp. Biol.* 225, jeb196725. 10.1242/jeb.196725 35188211

[JEB246186C48] Pamenter, M. E. and Powell, F. L. (2016). Time domains of the hypoxic ventilatory response and their molecular basis. *Comp. Physiol.* 6, 1345-1385. 10.1002/cphy.c150026 PMC4934681 27347896

[JEB246186C49] Pamenter, M. E., Hogg, D. W., Ormond, J., Shin, D. S., Woodin, M. A. and Buck, L. T. (2011). Endogenous GABA_A_ and GABA_B_ receptor-mediated electrical suppression is critical to neuronal anoxia tolerance. *Proc. Natl. Acad. Sci. USA* 108, 11274-11279. 10.1073/pnas.1102429108 21690381 PMC3131309

[JEB246186C50] Pamenter, M. E., Dzal, Y. A. and Milsom, W. K. (2015). Adenosine receptors mediate the hypoxic ventilatory response but not the hypoxic metabolic response in the naked mole rat during acute hypoxia. *Proc. Biol. Sci.* 282, 20141722. 10.1098/rspb.2014.1722 25520355 PMC4298202

[JEB246186C51] Pamenter, M. E., Lau, G. Y., Richards, J. G. and Milsom, W. K. (2018). Naked mole rat brain mitochondria electron transport system flux and H^+^ leak are reduced during acute hypoxia. *J. Exp. Biol.* 221, jeb171397. 10.1242/jeb.171397 29361591

[JEB246186C52] Pamenter, M. E., Dzal, Y. A., Thompson, W. A. and Milsom, W. K. (2019). Do naked mole rats accumulate a metabolic acidosis or an oxygen debt in severe hypoxia? *J. Exp. Biol.* 222, jeb191197. 10.1242/jeb.191197 30573665

[JEB246186C53] Phillis, J. W., O'regan, M. H. and Perkins, L. M. (1992). Measurement of rat plasma adenosine levels during normoxia and hypoxia. *Life Sci.* 51, PL149-PL152. 10.1016/0024-3205(92)90363-T 1528087

[JEB246186C54] Polson, J. B., Krzanowski, J. J., Goldman, A. L. and Szentivanyi, A. (1978). Inhibition of human pulmonary phosphodiesterase activity by therapeutic levels of theophylline. *Clin. Exp. Pharmacol. Physiol.* 5, 535-539. 10.1111/j.1440-1681.1978.tb00707.x 215363

[JEB246186C55] Powell, F. L., Milsom, W. K. and Mitchell, G. S. (1998). Time domains of the hypoxic ventilatory response. *Respir. Physiol.* 112, 123-134. 10.1016/S0034-5687(98)00026-7 9716296

[JEB246186C56] Remler, M. P. and Marcussen, W. H. (1985). Bicuculline methiodide in the blood–brain barrier-epileptogen model of epilepsy. *Epilepsia* 26, 69-73. 10.1111/j.1528-1157.1985.tb05189.x 3971950

[JEB246186C57] Saito, H., Nishimura, M., Shinano, H., Makita, H., Tsujino, I., Shibuya, E., Sato, F., Miyamoto, K. and Kawakami, Y. (1999). Plasma concentration of adenosine during normoxia and moderate hypoxia in humans. *Am. J. Respir. Crit. Care. Med.* 159, 1014-1018. 10.1164/ajrccm.159.3.9803100 10051286

[JEB246186C58] Swoap, S. J., Iliff, B. W. and Le, S. (2012). Adenosine, AMP, and daily torpor. In *Living in a Seasonal World*, (ed. T. Ruf, C. Bieber, W. Arnold and E. Millesi), pp. 337-339. Springer.

[JEB246186C59] Teppema, L. J. and Dahan, A. (2010). The ventilatory response to hypoxia in mammals: mechanisms, measurement, and analysis. *Physiol. Rev.* 90, 675-754. 10.1152/physrev.00012.2009 20393196

[JEB246186C60] Thomas, H. G., Swanepoel, D. and Bennett, N. C. (2016). Burrow architecture of the Damaraland mole-rat (*Fukomys damarensis*) from South Africa. *Afr. Zool.* 51, 29-36. 10.1080/15627020.2015.1128355

[JEB246186C61] Thorley, J. and Clutton-Brock, T. H. (2019). A unified-models analysis of the development of sexual size dimorphism in Damaraland mole-rats, *Fukomys damarensis*. *J. Mammal.* 100, 1374-1386. 10.1093/jmammal/gyz082

[JEB246186C62] Tomasco, I. H., Del Rio, R., Iturriaga, R. and Bozinovic, F. (2010). Comparative respiratory strategies of subterranean and fossorial octodontid rodents to cope with hypoxic and hypercapnic atmospheres. *J. Comp. Physiol. B* 180, 877-884. 10.1007/s00360-010-0465-y 20352232

[JEB246186C63] Tupone, D., Madden, C. J. and Morrison, S. F. (2013). Central activation of the A_1_ adenosine receptor (A1AR) induces a hypothermic, torpor-like state in the rat. *J. Neurosci.* 33, 14512-14525. 10.1523/JNEUROSCI.1980-13.2013 24005302 PMC3761054

[JEB246186C64] Ueno, S., Bracamontes, J., Zorumski, C., Weiss, D. S. and Steinbach, J. H. (1997). Bicuculline and gabazine are allosteric inhibitors of channel opening of the GABA_A_ receptor. *J. Neurosci.* 17, 625-634. 10.1523/JNEUROSCI.17-02-00625.1997 8987785 PMC6573228

[JEB246186C65] Vandewint, A. L., Zhu-Pawlowsky, A. J., Kirby, A., Tattersall, G. J. and Pamenter, M. E. (2019). Evaporative cooling and vasodilation mediate thermoregulation in naked mole-rats during normoxia but not hypoxia. *J. Therm. Biol.* 84, 228-235. 10.1016/j.jtherbio.2019.07.011 31466758

[JEB246186C66] Veng-Pedersen, P., Brashear, R. E. and Karol, M. D. (1983). Theophylline blood–brain barrier transfer kinetics in dogs. *J. Pharm. Sci.* 72, 951-953. 10.1002/jps.2600720830 6620157

[JEB246186C67] Wong-Riley, M. T. T., Liu, Q. and Gao, X. (2019). Mechanisms underlying a critical period of respiratory development in the rat. *Respir. Physiol. Neurobiol.* 264, 40-50. 10.1016/j.resp.2019.04.006 30999061 PMC6564680

[JEB246186C68] Zhang, S. Y. and Pamenter, M. E. (2019a). Fossorial Damaraland mole rats do not exhibit a blunted hypercapnic ventilatory response. *Biol. Lett.* 15, 20190006. 10.1098/rsbl.2019.0006 30862308 PMC6451387

[JEB246186C69] Zhang, S. Y. and Pamenter, M. E. (2019b). Ventilatory, metabolic, and thermoregulatory responses of Damaraland mole rats to acute and chronic hypoxia. *J. Comp. Physiol. B* 189, 319-334. 10.1007/s00360-019-01206-y 30725174

[JEB246186C70] Zhang, M., Clarke, K., Zhong, H., Vollmer, C. and Nurse, C. A. (2009). Postsynaptic action of GABA in modulating sensory transmission in co-cultures of rat carotid body via GABA(A) receptors. *J. Physiol.* 587, 329-344. 10.1113/jphysiol.2008.165035 19029183 PMC2670048

[JEB246186C71] Zimmerman, B., Diebold, G., Galbraith, J., Whitmore, W., Okamoto, M., Robinson, J. B., Young, B. A., Murdoch, G., Mosenthin, R. and Christopherson, R. J. (2003). Effect of aminophylline on metabolic and thermoregulatory responses during hypothermia associated with cold exposure in lambs. *Can. J. Anim. Sci.* 83, 739-748. 10.4141/A03-013

[JEB246186C72] Zions, M., Meehan, E. F., Kress, M. E., Thevalingam, D., Jenkins, E. C., Kaila, K., Puskarjov, M. and Mccloskey, D. P. (2020). Nest carbon dioxide masks GABA-dependent seizure susceptibility in the naked mole-rat. *Curr. Biol.* 30, 2068-2077.e4. 10.1016/j.cub.2020.03.071 32359429

